# A Longitudinal Study of the Impact of Social Deprivation and Disease Severity on Employment Status in the UK Cystic Fibrosis Population

**DOI:** 10.1371/journal.pone.0073322

**Published:** 2013-08-23

**Authors:** David C. Taylor-Robinson, Rosalind Smyth, Peter J. Diggle, Margaret Whitehead

**Affiliations:** 1 Department of Public Health and Policy, University of Liverpool, Liverpool, United Kingdom; 2 Institute of Child Health, UCL, London, United Kingdom; 3 Institute of Infection and Global Health, University of Liverpool, Liverpool, United Kingdom; University of Colorado, Denver, United States of America

## Abstract

**Background:**

People with Cystic Fibrosis (CF) in the UK and elsewhere are increasingly surviving into adulthood, yet there is little research on the employment consequences of having CF. We investigated, for the first time in a UK-wide cohort, longitudinal employment status, and its association with deprivation, disease severity, and time in hospital.

**Methods:**

We did a longitudinal registry study of adults with CF in the UK aged 20 to 40 (3458 people with 15,572 observations between 1996 and 2010), using mixed effects models.

**Results:**

Around 50% of adults with CF were in employment. Male sex, higher lung function and body mass index, and less time in hospital were associated with improved employment chances. All other things being equal, being in the most deprived quintile was associated with a reduction of employment prevalence of 17.6 percentage points compared to the prevalence in the least deprived quintile. Having poor lung function was associated with a reduced employment prevalence of 7.2 percentage points compared to the prevalence for people with relatively good lung function. Acting synergistically, deprivation modifies the effect of lung function on employment chances – poor lung function in the least deprived group was associated with a 3 percentage point reduction in employment chances, while poor lung function in the most deprived quintile was associated with a 7.7 point reduction in employment chances.

**Conclusions:**

Greater deprivation, disease severity, and time in hospital are all associated with employment chances in adults with CF. Furthermore, our analysis suggests that deprivation amplifies the harmful association of disease severity on employment. Future studies should focus on understanding and mitigating the barriers to employment faced by people with CF.

## Introduction

Cystic fibrosis (CF) is the commonest life-limiting inherited disease among Caucasian populations, with most patients dying prematurely from respiratory failure. Children with CF in the UK and other high-income countries are usually diagnosed in the first year of life [1], and subsequently require intensive support from family and healthcare services. People with CF in the UK and elsewhere are increasingly surviving into adulthood, with the median age of survival estimated to be over fifty years for a person born in this century [2]. One implication of this improved survival, is that increasing attention needs to be paid to the experiences of people with CF when they reach adulthood and enter employment.

Employment is one of the “social determinants” of health [3]. Work influences health in a number of ways; it provides income to meet material needs, but also fulfils critical psycho-social functions, increasing self-worth, sense of identity and opportunities for social interaction. Numerous studies have identified unemployment as a potent risk factor for poor health, and equally, poor health can lead to reduced employment chances [4], [5]. People with chronic illnesses face numerous barriers to entering the labour market, and CF provides a case in point. Factors related to disease severity, such as reduced lung function may restrict employment choices for adults with CF, and the treatment burden further compounds this: adults with CF are generally expected to perform physiotherapy regularly and there are the added demands of taking large numbers of therapies, including frequent visits to hospital [6].

Despite these potential challenges, the evidence about patterns of employment for adults with CF is limited [7], and mainly based on cross-sectional studies of single centres, which cannot delineate the relationships between chronic illness and employment outcomes or whether these relationships indicate causality. Furthermore, Edwards et al [8], adopting the social model of disability, have criticised the approach taken to understanding employment outcomes in CF. They point out that most of the research, to date, has been restricted to the effects of disease severity on employment chances, ignoring the significant structural and societal barriers to employment for people with chronic illness.

Exploring inequalities by socio-economic status (SES) in employment outcomes in people with CF is a key step in understanding how health and social inequalities are generated and perpetuated. Because CF is a classically inherited genetic disease, unlike most chronic diseases, there is no difference in the incidence of the condition with socioeconomic status [9], [10]. However, inequalities develop over the course of people’s lives, as a consequence of having the disease. Informed by Diderichsen’s analytic framework of the pathways from social context to health outcomes [11], we have demonstrated that in the UK CF population there are clinically important differences in growth, and lung function by deprivation, which are evident early on in children’s lives [9], [10]. Furthermore, people with CF from socio-economically disadvantaged backgrounds die at a younger age than those in more advantaged social positions in the UK and the US [12]–[15]. The social patterning of outcomes in cystic fibrosis implies that the mechanism of *differential exposure* to social and environmental risk factors is playing an important role in influencing outcomes [3], .

Building on these findings, the next step is to look for any “differential social consequences” of ill-health in the context of CF [11]. Our aims in this study were to explore the association of prior deprivation, disease severity, and time in hospital on longitudinal employment chances in people with CF and to investigate whether changes in lung function have differential effects on employment chances by deprivation (‘*differential social consequences’ in Diderichsen’s model*). For instance, is poor lung function in CF more damaging to employment chances in people from more disadvantaged areas? We undertook a longitudinal population level registry study of employment status in adults with CF in the UK to address this question.

## Methods

### Ethics statement

NHS research ethics approval (Huntingdon Research Ethics Committee 07/Q0104/2) has been granted for the collection of data into the UK database. Each patient provided written informed consent for collection of data in the registry, and for use of anonymised data in research. The CF Trust database committee approved the use of anonymised data in this study, under the terms of the NHS ethics approval.

### Design, setting and data source

We undertook a longitudinal retrospective cohort study of annual review data on individuals between the ages of 20 and 40 with at least one outcome measurement and a valid postal code in the UK CF Registry between 1996 and 2010. The UK CF Registry, co-ordinated by the Cystic Fibrosis Trust [17], [18], is maintained to a high standard of data quality, and includes nearly all people with CF in the UK population [19], with an estimated coverage of over 99% [9], and is therefore ideally suited to the study of prior exposures on subsequent employment outcomes across the whole socioeconomic spectrum in the UK society.

### Primary outcome and covariates

The primary longitudinal outcome was any employment (defined as “full” or “part-time” as specified in the drop-down menu in the UK CF registry) in the preceding year (yes or no) recorded at annual review. The primary exposure measures were deprivation of small-area of residence, as commonly used in epidemiological studies in the UK [9], lung function (as measured by forced expiratory volume in one second -%FEV1), and time in hospital. Postcodes were used to derive Index of Multiple Deprivation (IMD) scores for the constituent UK countries [20] and each person was allocated to a deprivation quintile on the basis of first recorded postcode. These indices combine economic, social and housing indicators measured at the census into a composite deprivation score for small areas in the UK constituent countries. Baseline covariates in the analysis were: sex; genotype coded as the number of delta F508 alleles (0, 1 or 2); and year of birth. We adjusted for disease severity on the basis of degree of impaired lung function measured by %FEV1, as this measure is strongly predictive of survival [21], and deviation from expected Body Mass Index (as measured by Body Mass Index standard deviation score (BMI SD score). As a measure of time spent administering therapies, we included the number of intravenous (IV) therapy days in the past year in our analysis, further disaggregated into therapy days in hospital, and therapy days at home. We first fitted a model adjusted for age and the baseline covariates, which are unlikely to be in the causal path from SES to employment status. We then tested for the significance of adding disease severity measures, and service use measures, which may be in the causal path, and finally added deprivation score to the model. The logic model for the analysis is shown in Figure S1.

### Statistical Methods

Repeated measures on individuals are correlated, and this must be accommodated to obtain valid inferences. For a full description see the appendix S1. In brief, we applied advanced statistical methods that have been specifically developed for the analysis of longitudinal data, after Diggle et al, 2002 (24). Exploratory statistical analysis involved: fitting generalized additive models (GAMs) [22] to visualize the shape of associations; plotting empirical logits; and plotting stratified raw data. We then fitted generalised linear mixed models (GLMMs) to the data across the age range. These procedures model the log-odds of employment status as a linear function of the measured covariates and individual level random-effects, and adjust the standard errors of the regression parameters to take account of the correlation structure of the repeated measurements. We fitted sequential models adjusting for the covariates of interest, and estimated model parameters by maximum likelihood, using generalized likelihood ratio statistics to compare nested models, and Wald statistics to test hypotheses about model parameters [23]. These longitudinal analyses take into account drop-out due to death, and implicitly estimate the chances of employment in a hypothetical drop-out free population [24]. We present effect estimates as log-odds with confidence intervals, since odds ratios can be mis-interpreted when outcomes are common [25]. To aid interpretation, we display population-averaged employment chances in the plots, by averaging individual-level fitted values over the population.

## Results

The final dataset contained 3,458 people, with 15,098 person-years of follow-up, and data collected at 15,572 annual reviews. 1940 (56%) individuals had four or more follow-up measures (median 4, interquartile range 2 to 7). The baseline characteristics of individuals at first recorded entry into the cohort are shown in table 1.

**Table 1:** Characteristics of study population in UK CF Registry by employment status at baseline.

	Not in employment	Employed	Total
Number of adults with CF (%)	1845 (53.4)	1613 (46.6)	3458
Observations (%)	7287 (46.8)	8285 (53.2)	15572
Deprivation quintile 1 (least deprived)	295 (16)	344 (21.3)	639 (18.5)
Deprivation quintile 2	319 (17.3)	370 (22.9)	689 (19.9)
Deprivation quintile 3	357 (19.3)	354 (21.9)	711 (20.6)
Deprivation quintile 4	417 (22.6)	321 (19.9)	738 (21.3)
Deprivation quintile 5 (most deprived)	457 (24.8)	224 (13.9)	681 (19.7)
Number of F508 alleles:2 (%)	952 (51.6)	744 (46.1)	1696 (49)
Number of F508 alleles:1 (%)	632 (34.3)	616 (38.2)	1248 (36.1)
Number of F508 alleles:0 (%)	261 (14.1)	253 (15.7)	514 (14.9)
Female	856 (46.4)	672 (41.7)	1528 (44.2)
Non-white	54 (2.9)	27 (1.7)	81 (2.3)
Birth cohort 1959 – 1968	152 (8.2)	211 (13.1)	363 (10.5)
Birth cohort 1969 – 1978	409 (22.2)	573 (35.5)	982 (28.4)
Birth cohort 1979 – 1988	1203 (65.2)	780 (48.4)	1983 (57.3)
Birth cohort >1988	81 (4.4)	49 (3)	130 (3.8)
Median age at baseline (years) (IQR)	21 (20.4,24.5)	23 (20.7,29.3)	21.5 (20.5,27)
Median %FEV1 at entry (IQR)	61.8 (41.8,82.2)	68.4 (49.9,85.2)	65.3 (45.8,83.7)
%FEV1 >90 (normal)	286 (15.5)	302 (18.7)	588 (17)
%FEV1 >70 and <90 (mild)	457 (24.8)	458 (28.4)	915 (26.5)
% FEV1 >40 and <70 (moderate)	678 (36.7)	619 (38.4)	1297 (37.5)
% FEV1 <40 (severe)	424 (23)	234 (14.5)	658 (19)
Pseudomonas colonization at entry	1123 (60.9)	836 (51.8)	1959 (56.7)
Median BMI SDS at entry (IQR)	–0.6 (–1.4,0.1)	–0.4 (–1.2,0.4)	–0.5 (–1.3,0.3)
Died	223 (12.1)	115 (7.1)	338 (9.8)

**Figure 1:**
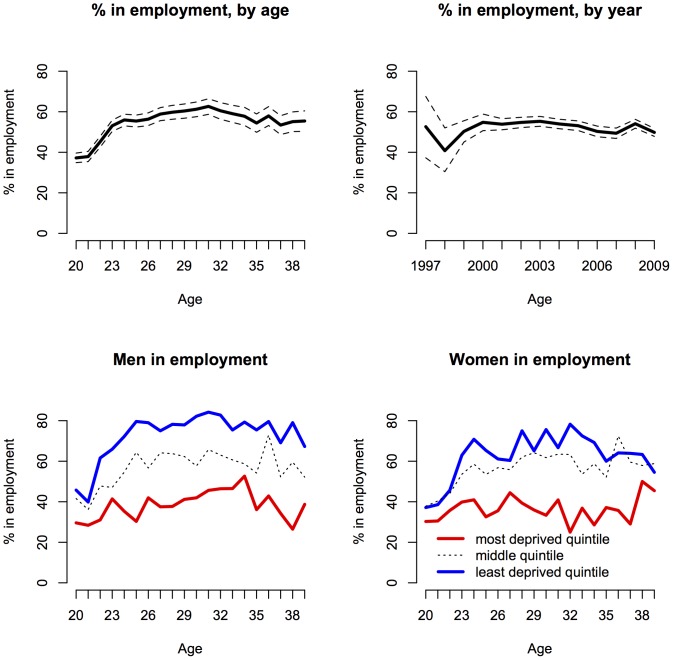
Overall employment prevalence by age and year of people with CF aged 20–40 in UK CF Registry. 95% confidence intervals (top row). Bottom row shows employment prevalence by age, stratified by deprivation quintile (most deprived quintile in red), for men and women.

At any one time, about 50% of the UK CF population were recorded as being in full or part-time employment for all ages, but patterns differed by age, sex, and deprivation status (Figure 1). Across the entire age range, men and women with CF from the most deprived quintile were much less likely to be in employment that their counterparts in the least deprived quintile (Figure 1).

**Figure 2:**
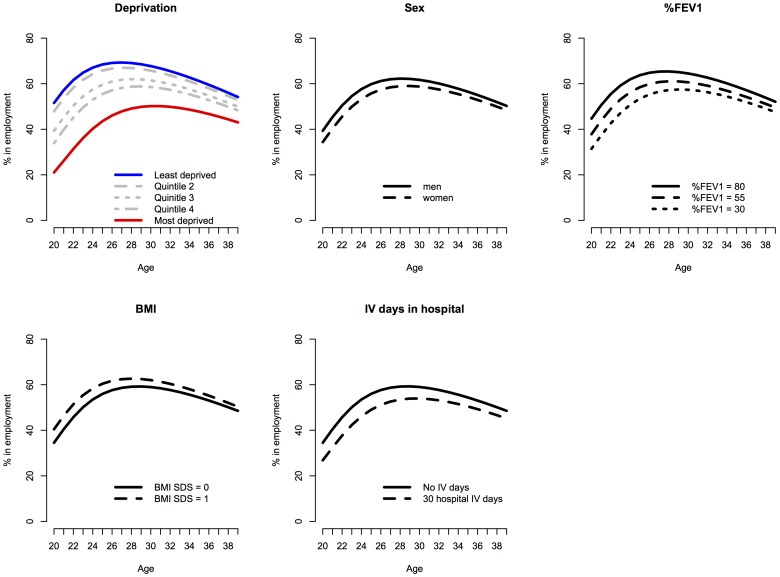
Longitudinal employment trajectory versus age of people with CF in UK CF Registry, by deprivation quintile, sex, %FEV1, BMI SD score and days in hospital. The lines show the final modelled longitudinal trajectories from the final interaction model (table 2, column 5), contrasting the adjusted effects of deprivation, sex, %FEV1, BMI SD score and days in hospital. These effects are plotted at the reference levels for the other covariates in the analysis.

Figure 2 illustrates the modelled independent population averaged relationship between deprivation, sex, lung function, weight and time in hospital, and employment chances for people with CF in the UK, on the basis of the final interaction model (table 2, column 5). There are significant age-related effects. The general pattern was for the proportion of people in employment to increase to around age 30, and decrease subsequently. Genotype and use of home intravenous therapy were not associated with employment chances in any of the analyses, and were dropped from the final models.

**Table 2:** Log odds for the final nested generalised mixed effects models (GLMMs) for the effects of disease severity, time in hospital and level of deprivation on employment chances.

	*Baseline*	*Baseline +severity*	*Baseline +Severity +Time in hospital*	*Baseline +Severity +Time in hospital +Deprivation*	*Baseline +Severity +Time in hospital +Deprivation* * *%FEV1*
Constant	0.663***	0.761***	0.997***	2.047***	2.058***
	(0.105)	(0.106)	(0.105)	(0.172)	(0.171)
age	0.128***	0.143***	0.144***	0.146***	0.146***
	(0.016)	(0.016)	(0.016)	(0.016)	(0.016)
agê2	–0.022***	–0.024***	–0.023***	–0.023***	–0.023***
	(0.002)	(0.002)	(0.002)	(0.002)	(0.002)
Birthyear	–0.057***	–0.059***	–0.046**	–0.039**	–0.039**
	(0.014)	(0.014)	(0.014)	(0.014)	(0.014)
Male/Female	0.446***	0.485***	0.443***	0.410**	0.401**
	(0.129)	(0.128)	(0.125)	(0.125)	(0.125)
Random intercept SD	(3.016)	(2.893)	(2.779)	(2.640)	(2.632)
Random slope SD	(0.450)	(0.451)	(0.438)	(0.442)	(0.441)
%FEV1		0.026***	0.020***	0.021***	0.013**
		(0.002)	(0.002)	(0.002)	(0.005)
BMI SDS score		0.163***	0.121**	0.106*	0.104*
		(0.043)	(0.043)	(0.043)	(0.043)
Hospital IV days			–0.024***	–0.023***	–0.023***
			(0.002)	(0.002)	(0.002)
Deprivation quintile 2/1				–0.270	–0.279
				(0.202)	(0.202)
Deprivation quintile 3/1				–0.967***	–0.976***
				(0.200)	(0.200)
Deprivation quintile 4/1				–1.422***	–1.427***
				(0.198)	(0.198)
Deprivation quintile 5/1				–2.650***	–2.663***
				(0.207)	(0.207)
Deprivation quintile 2/1 x%FEV1					0.010
					(0.007)
Deprivation quintile 3/1 x%FEV1					0.010
					(0.006)
Deprivation quintile 4/1 x%FEV1					0.006
					(0.006)
Deprivation quintile 5/1 x%FEV1					0.016*
					(0.007)
Log-likelihood	–7885	–7720	–7645	–7548	–7545
N	15572	15430	15430	15430	15430
Groups	3458	3451	3451	3451	3451
Baseline variance explained (%)	-	7.9	15.1	23.3	23.8

* *p*<0.05, ^**^ *p*<0.01, ^***^ *p*<0.001.

Standard errors in parentheses.

Of the covariates in the model, deprivation status explained more of the variance, and there was a dose-response relationship, in that the greater the level of deprivation the lower the chances if employment (figure 2, table 2). People in the most deprived quintile were less likely to be in employment, after adjusting for disease severity, compared to their more advantaged counterparts (log-odds –2.66 95%CI –3.1 to –2.26, comparing the most to the least deprived quintile). For men with a middling level of lung function (%FEV1 of 60) at the age of 30, this equates to 67.7% employment in the least deprived quintile, compared to 50.2% in the most deprived, a difference of 17.6 percentage points (figure 2). Comparing a population with relatively good lung function (a%FEV of 80), to one with poor lung function (a%FEV1 of 30), with all other things being equal (i.e. deprivation quintile 3, male sex), at the age of 30 there was a difference of 7.2 percentage points in employment chances (log-odds –0.63 95%CI –1.1 to –0.15 comparing poor to good lung function (figure 2).

Men were more likely than women to be in employment (log-odds 0.40 95%CI 0.16 to 0.64 in adjusted model), which corresponds to 61.7% employment in men, compared to 58.7% employment in women at age 30, for people with a%FEV1 of 60, in the middle deprivation quintile – a difference of 3 percentage points. People with better lung function were more likely to be in employment, and this followed a monotone dose response relationship. Higher BMI was associated with improved employment chances (log-odds 0.1 95%CI 0.020 to 0.188 per 1 unit increase in BMI SD score), and more days in hospital were associated with lower employment chances (log-odds –0.023 95%CI –0.027 to –0.019, per day in hospital).

**Figure 3:**
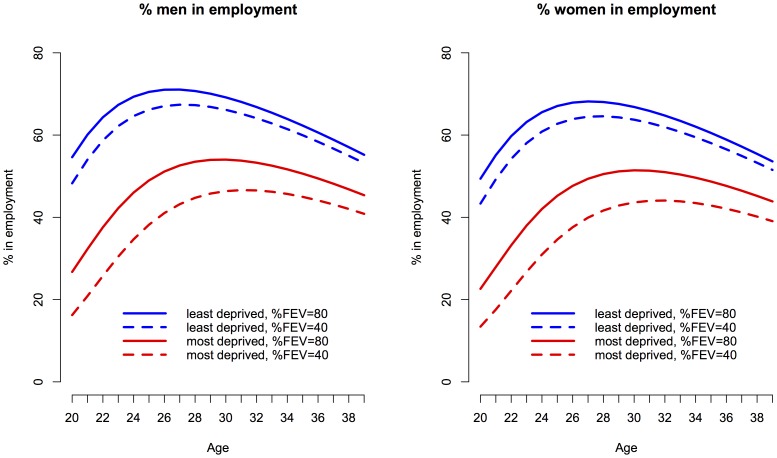
Longitudinal employment trajectory versus age, demonstrating the interaction between deprivation and lung function. The lines show the final modelled longitudinal trajectories from the interaction model (table 2, column 5), contrasting the adjusted effects of deprivation, and %FEV1.

Figure 3 illustrates the interactive effect of degree of disease severity (as measured by level of lung function) and social deprivation on population averaged employment chances in men and women. In the final model, a composite test for interaction between level of lung function and deprivation quintile was not significant (p = 0.2), but the contrast between the most deprived quintile and level of lung function was significant (log-odds 0.016 95% CI 0.0028 to 0.03, per unit increase in %FEV1, p = 0.018) (table 2). This suggests that having poor lung function was more damaging to the employment chances of the most disadvantaged quintile than for the least disadvantaged. At age 30, for example, poor lung function in men in the least deprived quintile was associated with 3.1 percentage points lower employment chances than for their counterparts in the same quintile with good lung function (66.1% employment prevalence compared with 69.2%). For men in the most deprived quintile, however, there is 7.7 percentage points difference in employment chances between those with poor and good lung function (46.4% compared with 54.1%).

## Discussion

We undertook a longitudinal registry-based study of employment status in the UK CF population, and found that lower social deprivation, male sex, higher level of lung function and BMI, and less time in hospital were associated with improved employment chances. All other things being equal, being deprived was associated with lower employment chances (17.6 percentage points lower than least deprived quintile). Having poor lung function was also associated with lower employment chances (7.2 percentage points lower in people with poor versus good lung function). When people with CF have a double burden of high deprivation and poor lung function, however, the impact on employment chances is magnified. In other words,, deprivation appears to modify the effect of lung function on employment chances - poor lung function is more harmful to employment chances in people living in the most deprived areas, compared to the least. As people are living longer, healthier lives with CF, it is more important than ever for strategies to promote employment to focus on the broader societal barriers to engagement in the workforce for people with CF, rather than taking a narrow ‘impairment’ focus solely on the impact of disease severity on employment chances, as critiqued by Edwards et al [8].

Key strengths of this study include the population-wide coverage of the UK CF registry, the high quality of the data, the longitudinal analysis, and the theoretical approach that responds to previous criticisms of the illness-focussed approach to understanding employment outcomes in CF. Our findings are particularly relevant to the UK population, but could be cautiously generalised to other high-income countries. There are limitations: it relies on retrospective, routinely collected data and although we used a standard, fine-grained measure of deprivation of area of residence, employed widely in epidemiological studies in the UK as a measure of socio-economic status (SES) [9], [26], [27], it was not possible to separate effects of socioeconomic circumstances operating at the individual and area level. There is thus the possibility of ecological bias, and this limits the possibilities to disentangle the mechanisms by which socioeconomic disadvantage operates. However, this possibility is minimized using the established Index of Multiple Deprivation methodology in the UK [9]. Secondly, we only had valid postcodes on 90% of the sample, though our sample size was large, with no pronounced gradient in the proportion of patients with valid postcodes by deprivation quintile i.e. missing postcode information was unrelated to deprivation. Finally the analysis did not include data for people aged over 40 years, because less than 5% of the annual clinical reviews occurred in patients over 40 years, so, although including these data would extend the age range for the analysis considerably, there would be small numbers.in the over-40 age-range.

A recent review concluded that further research on CF and employment is necessary to improve occupational outcomes [7]. The systematic review identified nine studies that have looked at the relationship between CF and employment status, all of which were small, and based at one or two care centres only. Six studies reported employment rates, all of around 50%. This systematic review did not include the largest study to date, by Walters et al [28], which was a cross-sectional questionnaire survey of 1052 adults over 16 years of age with CF in the UK in 1990. Walters et al found that 55% of responders were working, whilst of those not employed, half gave ill health as the reason [28]. In our study we find that the annual employment rate among CF adults in the UK appears to have remained unchanged between 50% and 55% over the last decade. Five previous studies in the systematic review analysed the association between disease severity, as measured by lung function, and employment status, but with no analysis by SES. The results were mixed: three studies conducted in the US [29], Canada [30] and Australia [31] concluded that lung function was not related to employment status, and two from the US [32] and Belgium [33] suggested that it was related. The studies by Burker et al. [29], Gillen et al. [32], and Hogg et al. [31] suggested that frequency of hospital admissions, demographic variables, mental health, and education level, may also influence employment status.

Our study suggests that disease severity and time in hospital influence employment chances in the UK CF population, but these effects are not as large as one might have predicted. It is evident that people with significant respiratory impairment continue to work, and disease severity alone does not predict employment outcomes. Furthermore, there is a large amount of variability between individuals with similar characteristics (large random effects), which suggests that there are other important factors related to employment status that we have been unable to account for in our study. Highest educational attainment is one such factor, but these data were available only for 60% of the individuals in the UK CF Registry. Adjusting for educational attainment did not change any of the substantive effects in our analysis, though it did reduce the random effects variance further (table S1). Other studies have also suggested that individual psychological factors and education status are correlated with employment status in CF [29].

These previous studies on employment chances in people with CF tend to portray CF as a ‘serious illness’, which causes employment problems. In contrast, Edward et al explored the employment experiences of adults with CF from a social model perspective. They demonstrated barriers to employment that were similar to those experienced by other disabled people, as well as barriers related to the ‘impairment effects’ of CF, and concluded that adults with CF have valuable perspectives to contribute to social model analysis and the development of employment-related policy solutions [8]. Our results not only corroborate, but also extend these observations, by demonstrating the interaction between disease severity related factors, and deprivation.

Our findings add to the extensive literature on the inter-relationship between chronic illness, socioeconomic status, and employment opportunities [34]. In the UK in 2005 the age standardized employment rate for people of working age (25–59) was 80% in healthy women, compared to 50% in those with limiting long-standing illness (LLSI), and 93% compared to 59% in men [34]. Furthermore there was a striking social gradient for those with LLSI - the prevalence of employment was 66% in highly educated women with LLSI compared to 18% in those with low education, and in men 72% compared to 30% [35]. In our study, the unadjusted prevalence of employment was around 60% in the most affluent quintile, compared to 30% in the most deprived quintile in women, and 70% versus 30% in men (figure 1).

Low employment in people with CF is a serious concern. Despite there being no difference in incidence of CF by socioeconomic status (SES), there are important differences in outcomes such as growth and lung function, and ultimately survival, in people with CF by SES in the UK and US [9], [12], [15]. Being out of work increases the risk of poverty and social exclusion, and is likely to further damage the health of the most disadvantaged people with CF. In this study we have demonstrated *differential social consequences of illness* in the context of CF, by which people with the double burden of chronic illness and low SES are more likely to be excluded from the labour market. We speculate that this may be an important pathway for the amplification of health inequalities in CF, whereby disadvantage builds on disadvantage. It is of particular concern that the most disadvantaged women have the poorest employment chances, since female sex is also an important risk factor for poor survival in CF [15].

In conclusion, this study has identified important longitudinal inequalities in employment outcomes in people with CF in the UK. Future studies should focus on policy interventions that would help overcome the extra burden of adverse consequences of CF faced by patients living in disadvantaged circumstances.

## Supporting Information

Figure S1 Logic model to inform analysis of employment status.
(PDF)

Table S1 Log odds for the final generalised mixed effects models (GLMMs), with added educational variable.
(DOCX)

Appendix S1 Supplementary text.
(DOCX)
