# Supplementary figures and images for: A Longitudinal Study of the Impact of Social Deprivation and Disease Severity on Employment Status in the UK Cystic Fibrosis Population
Source: PLoS One. 2013 Aug 23;8(8):e73322. doi: 10.1371/journal.pone.0073322 (PMC3751887; doi:10.1371/journal.pone.0073322)

**Figure S1: Logic model to inform analysis of employment status**

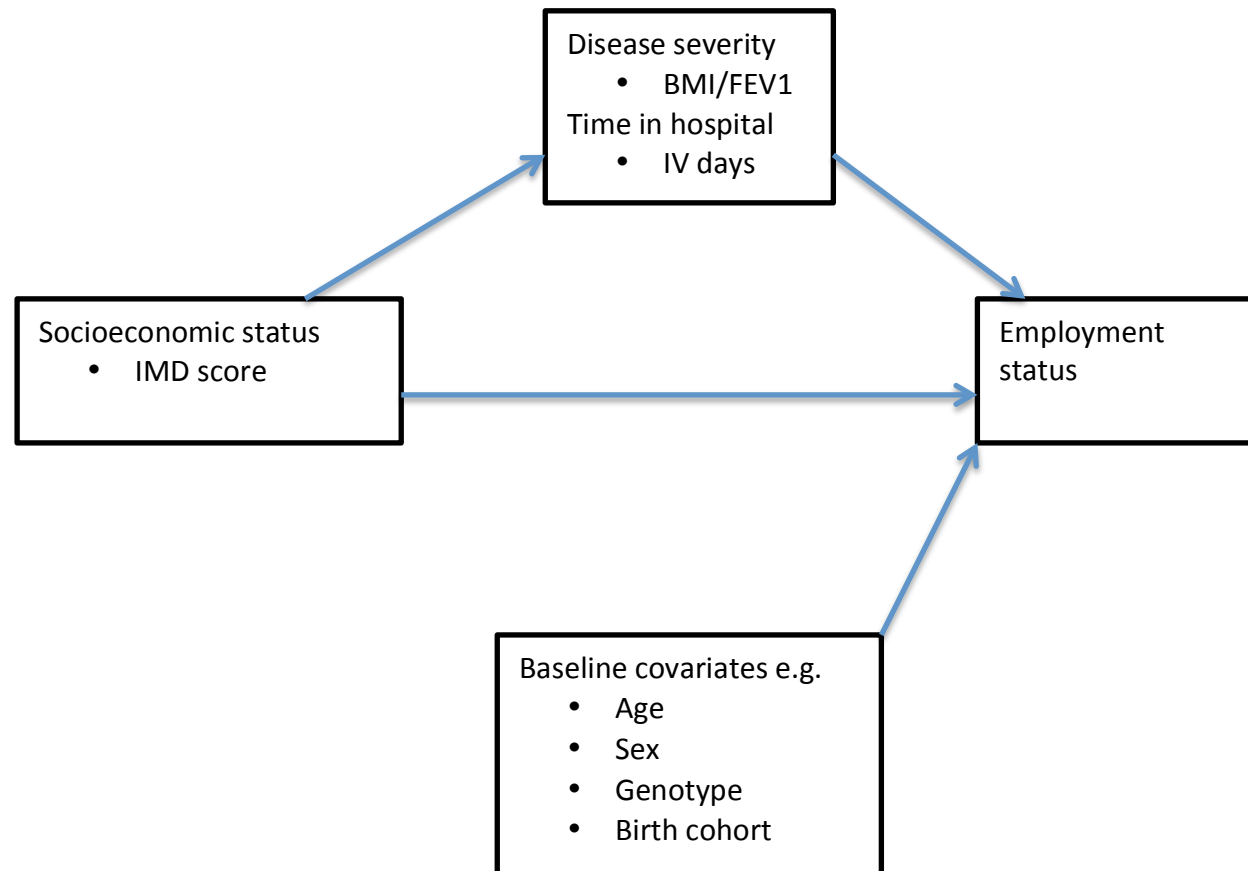

Supplement: Figure S1 — Logic model to inform analysis of employment status. (PDF) [file pone.0073322.s001.pdf]
