# Supplementary material for: A Longitudinal Study of the Impact of Social Deprivation and Disease Severity on Employment Status in the UK Cystic Fibrosis Population
Source: PLoS One. 2013 Aug 23;8(8):e73322. doi: 10.1371/journal.pone.0073322 (PMC3751887; doi:10.1371/journal.pone.0073322)
Supplement: Table S1 — Log odds for the final generalised mixed effects models (GLMMs), with added educational variable. (DOCX) [file pone.0073322.s002.docx]

**Table S1: Log odds for the final generalised mixed effects models (GLMMs), with added educational variable**

|  | *Baseline +Severity +Time in hospital +Deprivation*%FEV1* | *Plus education variable (NB models not nested)* |
| --- | --- | --- |
| Constant | 2.05821*** | -0.73603 |
|  | (0.17127) | (0.43094) |
| Deprivation quintile 2/1 | -0.27946 | -0.21829 |
|  | (0.20153) | (0.23348) |
| Deprivation quintile 3/1 | -0.97564*** | -0.70394** |
|  | (0.19987) | (0.24049) |
| Deprivation quintile 4/1 | -1.42676*** | -0.99734*** |
|  | (0.19752) | (0.23896) |
| Deprivation quintile 5/1 | -2.66327*** | -1.98129*** |
|  | (0.20678) | (0.25776) |
| %FEV1 | 0.01257** | 0.01111 |
|  | (0.00487) | (0.00579) |
| Hospital IV days | -0.02309*** | -0.02226*** |
|  | (0.00209) | (0.00282) |
| BMI SDS | 0.10406* |  |
|  | (0.04304) |  |
| age | 0.14598*** | 0.21636*** |
|  | (0.01616) | (0.02064) |
| age^2 | -0.02338*** | -0.02087*** |
|  | (0.00164) | (0.00212) |
| Birthyear | -0.03949** | -0.03994* |
|  | (0.01427) | (0.01826) |
| Male/Female | 0.40087** | 0.45083** |
|  | (0.12499) | (0.15167) |
| Deprivation quintile 2/1 x %FEV1 | 0.01031 | 0.00613 |
|  | (0.00661) | (0.00797) |
| Deprivation quintile 3/1 x %FEV1 | 0.00979 | 0.00676 |
|  | (0.00641) | (0.00819) |
| Deprivation quintile 4/1 x %FEV1 | 0.00581 | 0.01120 |
|  | (0.00638) | (0.00796) |
| Deprivation quintile 5/1 x %FEV1 | 0.01642* | 0.01814* |
|  | (0.00695) | (0.00881) |
| Ranef - id | 6.92797 | 6.05039 |
|  | (2.63210) | (2.45980) |
| Ranef - | 0.19489 | 0.18887 |
|  | (0.44146) | (0.43460) |
| Highest education: 2/1 (1 = “less than high school”) |  | 2.08872*** |
|  |  | (0.40422) |
| Highest education: 3/1 |  | 3.09579*** |
|  |  | (0.44268) |
| Highest education: 4/1 |  | 3.21147*** |
|  |  | (0.41106) |
| Highest education: 5/1 |  | 2.76440*** |
|  |  | (0.39619) |
| Log-likelihood | -7545.11112 | -4606.94109 |
| Deviance | 15090.22224 | 9213.88218 |
| AIC | 15128.22224 | 9257.88218 |
| BIC | 15273.45955 | 9415.17414 |
| N | 15430 | 9411 |
| Groups | 3451 | 2008 |
