# Supplementary material for: A Longitudinal Study of the Impact of Social Deprivation and Disease Severity on Employment Status in the UK Cystic Fibrosis Population
Source: PLoS One. 2013 Aug 23;8(8):e73322. doi: 10.1371/journal.pone.0073322 (PMC3751887; doi:10.1371/journal.pone.0073322)
Supplement: Appendix S1 — Supplementary text. (DOCX) [file pone.0073322.s003.docx]

**Online data supplement**

**A longitudinal study of the impact of social deprivation and disease severity on employment status in the UK cystic fibrosis population**

*David Taylor-Robinson, Rosalind Smyth, Peter Diggle, Margaret Whitehead*

# Methods

***Design, setting and data source***

Data are routinely collected in a standardized fashion at over 50 British cystic fibrosis specialist centres. Patients attending the British centres are seen in the outpatient clinic for a comprehensive annual review, including evaluation of clinical status, pulmonary function, microbiology of lower respiratory tract secretions, and use of CF major CF related therapies.

***Primary outcome and covariates***

Pulmonary function tests were performed according to international recommendations[[1](#_ENREF_1)], measuring forced expiratory volume in one second (%FEV_1_), expressed as a percentage of predicted values for sex and height using reference equations from Wang or Hankinson[[2](#_ENREF_2),[3](#_ENREF_3)].

The indices of multiple deprivation in the UK are widely used as measures of SES in epidemiological studies[[4-6](#_ENREF_4)] and are recommended for tracking health inequalities in UK government statistics[[7](#_ENREF_7)]. Indices of multiple deprivation combine economic, social and housing indicators measured at the census into a composite deprivation score for small areas in the UK constituent countries[[8](#_ENREF_8)]. There were 41500 of these small areas in the UK, containing on average 1400 people (range 500-3700). All of these small areas were ranked on the basis of the continuous deprivation score, and then divided into quintiles, providing the following approximate cut-off points for normative deprivation quintiles: <8.31; 8.32 to 13.81; 13.82 to 21.20; 21.21 to 34.11, >34.11. The IMD methodology allows much finer resolution than analyses using ZIP codes in the USA, which contain on average 30 000 people[[9](#_ENREF_9)].

***Statistical Methods***

Exploratory analysis involved using generalized additive models (GAMs)[[10](#_ENREF_10)] to visualize the shape of associations between covariates and employment chances and plotting empirical logits by age, sex, and deprivation quintile. We also stratified the raw data to look for evidence of an interaction between disease severity and deprivation, and then formally tested for this in the full dataset.

Repeated measures on individuals are correlated, and this must be accommodated to obtain valid inferences. To analyse the binary outcome we used a generalised linear mixed model[[11](#_ENREF_11)]. This specifies a logistic regression model for the effects of covariates on the probability of employment, but adjusts the standard errors of the regression parameters to take account of the correlation structure of the repeated measurements.

Specifically, denoting by *Y_ij_* the *j*th repeated binary outcome on the *i*th individual , *t_ij_* the age at the time of measurement and *p_ij_* the probability that *Y_ij_*=1, we assumed that *p_ij_* = *μ_ij_* + *U_i_* + *V_i_t_ij_*, where the *μ_ij_* are described by a multiple linear regression model and the (*U_i_,V_i_*) pairs are subject-specific intercepts and slopes, modelled as zero-mean bivariate Normally distributed random variables independently realised for different subjects, with means zero, variances $\sigma_{u}^{2}$ and $\sigma_{v}^{2}$ and correlation *ρ*.

We fitted generalised linear mixed models (GLMMs) to the data across the age range. These model the log-odds of employment status as a linear function of the measured covariates and individual level random-effects. Linear, quadratic and piecewise models for the mean trajectory were explored as informed by the GAMs.

We then fitted a model adjusted for age and the baseline covariates, and then tested for the significance of adding disease severity measures, and service use measures, and finally deprivation score. We estimated all model parameters by maximum likelihood, using generalised linear mixed effects models[[12](#_ENREF_12)]. These longitudinal models thus take into account drop-out due to death, and implicitly estimate the chances of employment in a drop-out free population, under the MAR (missing at random) assumption [[11](#_ENREF_11)]. We used generalized likelihood ratio statistics to compare nested models, and Wald statistics to test hypotheses about model parameters. Finally, as a robustness test, we undertook similar analyses using unemployment status at the primary outcome, and these produced complementary results. We present the effect estimates in terms of log-odds with confidence intervals, since odds ratios can be mis-interpreted when outcomes are common[[13](#_ENREF_13)]. To aid interpretation, we display population-averaged employment chances in the plots, by averaging individual-level fitted values over the population.

Statistical analysis was undertaken using R (version 2.9.2), and the lme4, survival and ggplot2 packages.

***Final dataset***

4062 people were recorded in the registry between the ages of >20 and <40years. 3495 of these had a valid postcode recorded and met the inclusion criteria. 3451 individuals had full data on all covariates in the final model.

**References:**

1. Miller MR, Hankinson J, Brusasco V, Burgos F, Casaburi R, et al. (2005) Standardisation of spirometry. Eur Respir J 26: 319-338.

2. Wang X, Dockery DW, Wypij D, Fay ME, Ferris BG, Jr. (1993) Pulmonary function between 6 and 18 years of age. Pediatr Pulmonol 15: 75-88.

3. Hankinson JL, Odencrantz JR, Fedan KB (1999) Spirometric reference values from a sample of the general U.S. population. Am J Respir Crit Care Med 159: 179-187.

4. Taylor-Robinson D, Agarwal U, Diggle PJ, Platt MJ, Yoxall B, et al. (2011) Quantifying the impact of deprivation on preterm births: a retrospective cohort study. PLoS One 6: e23163.

5. Semple MG, Taylor-Robinson DC, Lane S, Smyth RL (2011) Household tobacco smoke and admission weight predict severe bronchiolitis in infants independent of deprivation: prospective cohort study. PLoS One 6: e22425.

6. Bergen H, Hawton K, Waters K, Ness J, Cooper J, et al. (2012) Premature death after self-harm: a multicentre cohort study. Lancet 380: 1568-1574.

7. DH (2012) Improving outcomes and supporting transparency. Part 1: A public health outcomes framework for England, 2013-2016 <http://www.dh.gov.uk/prod_consum_dh/groups/dh_digitalassets/@dh/@en/documents/digitalasset/dh_132559.pdf> (accessed 3 December 2012).

8. ONS (2011) Indices of Deprivation across the UK <http://www.neighbourhood.statistics.gov.uk/dissemination/Info.do?page=analysisandguidance/analysisarticles/indices-of-deprivation.htm> (accessed 29th July 2011).

9. Krieger N, Waterman P, Chen JT, Soobader MJ, Subramanian SV, et al. (2002) Zip code caveat: bias due to spatiotemporal mismatches between zip codes and US census-defined geographic areas--the Public Health Disparities Geocoding Project. Am J Public Health 92: 1100-1102.

10. Hastie TJ, Tibshirani RJ (1990) Generalized additive models: Chapman & Hall/CRC.

11. Diggle P, Heagerty P, Liang K-Y, Zeger SL (2002) Analysis of Longitudinal Data (second edition). Oxford: Oxford University Press.

12. Laird NM, Ware JH (1982) Random-effects models for longitudinal data. Biometrics 38: 963-974.

13. Grimes DA, Schulz KF (2008) Making sense of odds and odds ratios. Obstet Gynecol 111: 423-426.
